# Cadmium Exposure and Incidence of Diabetes Mellitus - Results from the Malmö Diet and Cancer Study

**DOI:** 10.1371/journal.pone.0112277

**Published:** 2014-11-13

**Authors:** Yan Borné, Björn Fagerberg, Margaretha Persson, Gerd Sallsten, Niklas Forsgard, Bo Hedblad, Lars Barregard, Gunnar Engström

**Affiliations:** 1 Department of Clinical Sciences in Malmö, Lund University, Skåne University Hospital, Malmö, Sweden; 2 Sahlgrenska Center for Cardiovascular and Metabolic Research, Wallenberg Laboratory, Sahlgrenska University Hospital, Gothenburg, Sweden; 3 Department of Molecular and Clinical Medicine, University of Gothenburg, Gothenburg, Sweden; 4 Clinical Research Unit, Emergency Department, Skåne University Hospital, Malmö, Sweden; 5 Occupational and Environmental Medicine, Sahlgrenska University Hospital and University of Gothenburg, Gothenburg, Sweden; 6 Department of Clinical Chemistry, Sahlgrenska University Hospital, Gothenburg, Sweden; Virgen Macarena University Hospital, School of Medicine, University of Seville, Spain

## Abstract

**Background:**

Cadmium is a pollutant with multiple adverse health effects: renal dysfunction, osteoporosis and fractures, cancer, and probably cardiovascular disease. Some studies have reported associations between cadmium and impaired fasting glucose and diabetes. However, this relationship is controversial and there is a lack of longitudinal studies.

**Objectives:**

To examine prospectively whether cadmium in blood is associated with incidence of diabetes mellitus.

**Methods:**

The study population consists of 4585 subjects without history of diabetes (aged 46 to 67 years, 60% women), who participated in the Malmö Diet and Cancer study during 1991–1994. Blood cadmium levels were estimated from hematocrit and cadmium concentrations in erythrocytes. Incident cases of diabetes were identified from national and local diabetes registers.

**Results:**

Cadmium concentrations in blood were not associated with blood glucose and insulin levels at the baseline examination. However, cadmium was positively associated with HbA1c in former smokers and current smokers. During a mean follow-up of 15.2±4.2 years, 622 (299 men and 323 women) were diagnosed with new-onset of diabetes. The incidence of diabetes was not significantly associated with blood cadmium level at baseline, neither in men or women. The hazard ratio (4^th^ vs 1^st^ quartile) was 1.11 (95% confidence interval 0.82–1.49), when adjusted for potential confounders.

**Conclusions:**

Elevated blood cadmium levels are not associated with increased incidence of diabetes. The positive association between HbA1c and blood cadmium levels has a likely explanation in mechanisms related to erythrocyte turnover and smoking.

## Introduction

Cadmium is a non-essential toxic metal occurring in the environment naturally and as an industrial and agricultural pollutant. Cadmium is absorbed through the respiratory and digestive tracts via smoking, diet and occupational exposure in certain industries [1]. Smoking is associated with several-fold increases of blood cadmium [1]. In the past very high concentrations have been caused by various occupational exposures, however, this reason for high exposure is now rare in Sweden [2].Whole blood and urine concentrations of cadmium are valid biomarkers of exposure, irrespective of source. Most of the body burden of cadmium has a very long biological half-life (10–30 years). Blood cadmium is affected by the body burden as well as recent exposure [1]. Cadmium exposure has been associated with multiple adverse health effects including renal dysfunction [3], [4], [5], osteoporosis and fractures [6], [7], [8], [9], cancer [1], [10], [11], [12], [13], the development of atherosclerotic plaques [14] and cardiovascular disease (CVD) [15], [16].

Experimental studies have indicated that cadmium may have diabetogenic effects on pancreas, liver, adipose tissue and the adrenal gland models [17]. However, experimental data are conflicting and it has been shown that cadmium may also enhance the ratio of glucose-stimulated insulin release [18]. The Third National Health and Nutrition Examination Survey (NHANES 1988–1994) in the United States observed an association between cadmium exposure and pancreatic cancer and found support for a hypothesis that cadmium might be associated with increased risk of diabetes [19]. The authors found that the urinary cadmium level was associated with impaired fasting glucose (IFG) and diabetes in a cross-sectional study [19]. A suggestive mechanism was that cadmium might impair pancreatic production of insulin. The relationship between cadmium and diabetes in the NHANES study [19] has not been replicated in three population-based cross-sectional studies from Korea [20], Thailand [21] and Sweden [22]. However, to date there has not been any representative, sufficiently large population-based study that has examined prospectively if cadmium exposure is accompanied by increased risk of diabetes. Since diabetes is regarded as a major public health problem, it is important to identify possible risk factors. Hence, the purpose of the present study was to explore if elevated blood cadmium levels are associated with increased incidence of diabetes in middle-aged men and women from the general population.

## Methods

### Study population

The Malmö diet and cancer (MDC) is a prospective cohort study from the city of Malmö in southern Sweden [23], [24], [25]. During March 1991 to September 1996, 28 449 men (N = 11 246, born 1923–1945) and women (N = 17 203, born 1923–1950) attended a baseline examination. Participants underwent physical examination, sampling of peripheral venous blood, and filled out a questionnaire. A random sample of the MDC cohort, the MDC cardiovascular cohort (MDC-CV) (N = 6 103), was examined between October 1991 and February 1994, with the purpose of studying the epidemiology of carotid artery disease [26]. Of the 6 103 participants in the MDC-CV, 5 533 donated blood after fasting overnight, including measurements of fasting whole blood glucose, plasma insulin and hemoglobin A1c (HbA1c). Cadmium in blood could be analysed in 4 952 subjects (aged 46–67 years, 60% women). After excluding subjects with history of diabetes (n = 204) and missing values on waist circumference, smoking status, HbA1c or glucose, the study population included 4 585 subjects. Information on the Homeostasis Model Assessment (HOMA) index and insulin were available in 4 435 of the participants. Possible occupational exposure was evaluated from the subjects' job histories.

The study complies with the Declaration of Helsinki. All participants provided written informed consent, and the study was approved by the Lund University Ethics Review Committee.

### Measurements and definitions

Information on history of anti-diabetic medications and smoking habits were obtained from a self-administered questionnaire [23]. Waist circumference (in cm) was measured midway between the lowest rib margin and iliac crest. Subjects were categorized into current smokers, former smokers or never smokers.

### Laboratory measurements

Cadmium was analyzed in erythrocytes; the whole blood cadmium concentrations were calculated using erythrocyte concentrations adjusted for hematocrit. Erythrocyte concentrations of cadmium were analyzed using inductively coupled plasma mass spectrometry with an octopole reaction system (Agilent 7700x ICP-MS, Agilent Technologies). All samples were analyzed in three different rounds with two external quality control (QC) samples included. The results from all rounds versus recommended limits were 0.34±0.02 µg/L (N = 70) versus 0.32–0.40 µg/L and 5.7±0.18 µg/L (N = 70) versus 5.4–6.2 µg/L. The results were similar for the three different rounds. A comparison including 20 erythrocyte samples (range 0.2–0.96 µg/L) were made with another laboratory (Occupational and Environmental Medicine, Lund, Sweden). The results showed good agreement with a Pearson correlation coefficient of 0.99 and a slope of 1.04 (standard error 0.04).

HbA1c and whole blood glucose were measured according to standard procedures at the Department of Clinical Chemistry, Malmö University Hospital. HbA1c was measured by ion exchange chromatography; reference values were 3.9–5.3% in non-diabetic individuals. Insulin was measured by radioimmunoassay in mIU/L and the HOMA index was calculated as fasting insulin x glucose/22.5 [26]. Diabetes mellitus at baseline was defined as self-reported diabetes (according to the questionnaire), a fasting whole blood glucose ≥6.1 mmol/L [27], use of anti-diabetic medication or any recording in registers of diabetes prior to the baseline examination.

### Incidence of diabetes

All subjects were followed from the baseline examination until first diagnosis of diabetes, death, emigration from Sweden or December 31^st^, 2009, whichever came first. New-onset cases of diabetes in the MDC cohort were retrieved from several sources and have been described in detail previously. In short, incident diabetes was identified in the Malmö HbA1c register (MHR), the Swedish National Diabetes Register (NDR), the Swedish inpatient register, the Swedish outpatient register, the nationwide Swedish drug prescription register and the regional Diabetes 2000 register of the Skåne region [28], [29]. The diabetes diagnosis in NDR and the Diabetes 2000 register were based on established diagnostic criteria (fasting plasma glucose concentration of ≥7.0 mmol/L, measured on 2 different occasions). Incident diabetes cases in the MHR were defined as individuals who had at least two HbA1c recordings ≥6.0% with the Swedish Mono-S standardization system (corresponding to 7.0% according to the US National Glycohemoglobin Standardization Program) after the baseline examination.

### Statistical analysis

Blood cadmium, insulin, and HOMA index showed right-skewed distributions and were logarithmically transformed. Cadmium was categorized into sex-specific quartiles to adjust for the difference between men and women. Cross-sectional relations of cadmium quartiles to risk factors were assessed using one-way ANOVA for continuous variables and logistic regression for dichotomous variables. Multiple linear regression was used to assess the relationship between cadmium and HbA1c (dependent variable). Cox proportional hazards regression was used to examine the association between cadmium (in sex-specific quartiles) and incidence of diabetes. Potential confounders included age, waist circumference and smoking status. Hazard ratios (HR), with 95% confidence intervals (CI) were calculated. The fit of the proportional hazards model was checked visually by plotting the incidence rates over time and by entering time-dependent variables into the model. A subgroup analysis was performed for subjects with impaired fasting glucose (IFG) at the baseline examination (i.e. fasting whole blood glucose 5.6–6.1 mmol L^−1^). All analyses were performed using IBM SPSS version 20 (IBM Corp.).

## Results

### Baseline characteristics

Baseline characteristics in relation to the sex-specific quartiles of cadmium are presented in Table 1. HbA1c and current smoking were associated with blood level of cadmium in men and women. Only two subjects reported occupations with possible exposure to cadmium. Both had blood cadmium levels in the second quartile.

**Table 1:** Baseline Characteristics for Malmö Diet and Cancer (MDC-CV) cohort in relation to quartiles (Q1–Q4) of cadmium in blood.

	Quartiles of blood cadmiium				
MDC (N = 4585)	Q1 (n = 1145)	Q2 (n = 1147)	Q3 (n = 1147)	Q4 (n = 1146)	*P* value
**Men** (n = 1831)	n = 457	n = 458	n = 458	n = 458	
Blood cadmium range (µg/L)	0.01–0.15	0.15–0.24	0.24–0.51	0.51–5.07	
Age (years)	56.8±5.9	57.5±6.0	58.4±5.9	57.1±5.9	0.13
Waist circumference (cm)	92.2±9.4	94.0±10.0	93.3±9.5	91.7±10.5	0.25
Smoking status					<0.001
Current smoker (%)	3.7	6.6	18.8	83.4	
Former smoker (%)	36.3	59.0	60.0	14.0	
Never smoker (%)	60.0	34.5	21.2	2.6	
Glucose(mmol L^−1^)	5.1±0.9	5.1±0.8	5.1±0.8	5.2±0.9	0.48
HOMA	1.5	1.5	1.6	1.6	0.62
Insulin (mIU L^−1^)	1.9	1.9	1.9	1.9	0.60
HbA1c (%)	4.7±0.5	4.7±0.5	4.8±0.6	4.9±0.6	<0.001
**Women** (n = 2754)	n = 688	n = 689	n = 689	n = 688	
Blood cadmium range (µg/L)	0.02–0.18	0.18–0.27	0.27–0.50	0.50–4.83	
Age (years)	56.9±5.8	57.9±6.0	58.1±5.8	56.6±5.9	0.47
Waist circumference (cm)	76.7±9.9	76.4±9.6	77.3±10.2	76.3±10.0	0.83
Smoking status					<0.001
Current smoker (%)	3.5	4.2	14.4	81.7	
Former smoker (%)	28.3	32.7	37.0	10.5	
Never smoker (%)	68.2	63.1	48.6	7.8	
Glucose(mmol L^−1^)	4.9±0.6	4.9±0.6	4.9±0.7	4.9±0.6	0.41
HOMA	1.3	1.3	1.3	1.2	0.18
Insulin (mIU L^−1^)	1.8	1.8	1.8	1.8	0.997
HbA1c (%)	4.7±0.4	4.7±0.4	4.8±0.5	5.0±0.5	<0.001

All values are mean±standard deviation, unless otherwise stated. Values of insulin and HOMA are presented as median of log. HOMA, homeostatic model assessment. WC, waist circumference.

### Cadmium and baseline HbA1c

Baseline HbA1c was positively associated with cadmium (4.7% vs 4.9% for men and 4.7% vs 5.0% for women in the 1st compared to 4th quartile, p<0.001), Table 1. The significant results (p<0.001) remained after adjustment for age, waist circumference and smoking status, both in men (p = 0.033) and women (p<0.001). After adjustments for age and waist circumference, HbA1c was positively associated with cadmium in separate analyses of current smokers (p<0.001) and former smokers (p = 0.001), but not in never smokers (p = 0.953).

### Incidence of diabetes in relation to cadmium

A total of 622 individuals (299 men and 323 women) had diabetes during a mean follow-up of 15.2±4.2 years (range 0–18.2 years). The incidence of diabetes was 11.2 per 1000 person years in men and 7.6 per 1000 in women. In those who developed diabetes during the follow up, the mean time between the baseline examination and the diabetes diagnosis was 9.5±5.5 years. Incidence of diabetes was not significantly higher in subjects in the 4^th^ compared to the 1^st^ quartile of cadmium (HR: 1.14, 95% CI: 0.91–1.41) in the crude model. The results were similar in separate analyses of men and women. The results persisted after additional adjustments for age, waist circumference and smoking status, Table 2. In the adjusted model, age (HR per year: 1.02; CI: 1.01–1.04, p = 0.003) and waist circumference (HR per cm 1.06; CI: 1.05–1.06. p<0.001) were significantly associated with incident diabetes.

**Table 2:** Incidence of diabetes in relation to quartiles of blood cadmium concentrations at baseline in middle-aged men (n = 1831) and women (n = 2754).

MDC	Quartiles of cadmium				
	Q1	Q2	Q3	Q4	P, trend
Diabetes, n (per 1000 person-years)	156 (8.8)	144 (8.1)	160 (9.2)	162 (9.8)	
HR (CI)*	1.00	0.92 (0.74–1.16)	1.05 (0.84–1.31)	1.14 (0.91–1.41)	0.16
HR (CI)**	1.00	0.88 (0.70–1.11)	0.99 (0.79–1.25)	1.11 (0.82–1.49)	0.51
**Men** (n = 1831)	n = 457	n = 458	n = 458	n = 458	
Diabetes, n (per 1000 person-years)	74 (10.7)	70 (10.2)	76 (11.4)	79 (12.5)	
HR (CI)*	1.00	0.94 (0.68–1.31)	1.06 (0.77–1.46)	1.18 (0.86–1.61)	0.24
HR (CI)**	1.00	0.82 (0.59–1.14)	0.94 (0.67–1.32)	0.90 (0.59–1.38)	0.98
**Women** (n = 2754)	n = 688	n = 689	n = 689	n = 688	
Diabetes, n (per 1000 person-years)	82 (7.5)	74 (6.8)	84 (7.8)	83 (8.1)	
HR (CI)*	1.00	0.91 (0.66–1.24)	1.04 (0.77–1.41)	1.10 (0.81–1.50)	0.39
HR (CI)**	1.00	0.93 (0.68–1.27)	0.96 (0.70–1.31)	1.21 (0.81–1.82)	0.57

HR. hazards ratio; Cl, confidence interval.

* crude model; **model with adjustment of age, waist circumference and smoking status.

Subcategory analyses of never-smokers, former smokers and current smokers were performed. In never-smokers, the HR for diabetes was 1.05 (CI: 0.55–2.01) for 4^th^ vs 1^st^ quartile of cadmium in the adjusted model. In former smokers, the corresponding HR was 1.24 (CI: 0.75–2.04), and the HR was 0.71 (CI: 0.36–1.39) in current smokers.

### Impaired fasting glucose and cadmium

A total of 390 individuals had IFG at baseline. Mean cadmium was 0.51 µg/L in subjects with IFG and 0.46 µg/L in those with normal blood glucose (p = 0.09). A total of 154 (39.5%) individuals with IFG developed diabetes during the follow-up. The HR of diabetes in IFG subjects in the 4^th^ (vs 1^st^) quartile of cadmium was 0.80 (CI:0.51–1.24) after adjustment for age, waist circumference and smoking status.

## Discussion

In this prospective study we observed no significant association between cadmium exposure and incident diabetes, neither in men nor in women. Cadmium was also unrelated to incidence of diabetes in separate analyses of current-, former- and never-smokers. In addition, cadmium levels in blood were not associated with insulin, HOMA or glucose. The results remained non-significant after adjustments for confounding factors and we conclude that elevated blood cadmium levels are not associated with increased incidence of diabetes. The results are in accordance with those from two previous small prospective studies, one from Sweden performed in 64-year old women [22], and one from Thailand conducted in subjects who had been exposed to higher levels of cadmium [30]. The results are also in accordance with previous findings from several cross-sectional studies [15], [18], [20], [21], [22].

The concentrations of blood cadmium were low in this study; median was 0.24 µg/L for men and 0.27 µg/L for women. Tentatively the cadmium levels in the present study might have been too low to cause any diabetogenic effects. This is refuted by the results from previous studies in high exposure countries showing that cadmium levels which are 3 to 26 times higher [18], [20], [21], [30] than those of a typical Swedish exposure level [22] are not associated with the prevalence or incidence of diabetes. On the other hand, the cadmium exposure level in Sweden is sufficiently high to be associated with bone damage [7], [8], cancer [12], [13], and the development of atherosclerotic plaques [14]. Therefore we do not consider the moderate levels of cadmium levels in the present study to be an explanation of our negative findings.

Blood cadmium was associated with HbA1c but there was no significant relationship between cadmium and levels of blood glucose, serum insulin and HOMA. We and others have previously reported that cadmium concentrations in blood or urine are associated with HbA1c, but not with plasma insulin, blood glucose, measures of insulin resistance and pancreatic insulin production [20], [22]. Hence, there is discordance between the lack of association between the cadmium levels and direct measures of glucose and insulin metabolism and the strong relationship between cadmium and HbAlc. The underlying mechanisms were not investigated in the present study. However, available data indicate that the relationship between HbA1c and cadmium is related to the fact that both cadmium and HbA1c accumulate in the red cell, rather than to glucose metabolism and diabetes, and that smoking is also involved in this process. Firstly, the half-life of cadmium in erythrocytes is mainly related to their life time [31] and the HbA1c level increases with the erythrocyte life span [32]. Secondly, even if the average life span of erythrocytes is about 120 days, there is considerable variation between individuals [33]. The observation that blood cadmium and HbA1c were only associated in former and current smokers in the present study may be explained by the higher mean values of cadmium and much wider distribution of cadmium concentrations in ever smokers than in never smokers. At the same time smoking is *per s*e associated with increased HbA1c [34], which also hypothetically could be related to red cell life span [35]. Hence, it is reasonable to conclude that the relationship between cadmium and HbA1c has a likely explanation in mechanisms related to erythrocyte turnover and smoking, and that this cannot be explained by increased prevalence of diabetes.

### Limitation

One limitation of the present study is the lack of information on type of diabetes. Because the subjects of study were over 46 years old, it can be assumed that the majority of the incident cases developed type 2 diabetes, since type 1 diabetes usually has early onset [36]. Another question is whether the study cohort was representative for the general population. A previous study from the city of Malmö showed no substantial difference in baseline characteristics, such as smoking and obesity, between participants in the MDC and a health survey from the same city with 75% participation rate [37].

Regarding validation of end-points, new cases of diabetes were identified from several independent data sources. The hospital registers of out-and in-patients cover hospital visits in the whole country. The drug prescription register covers all filled prescriptions from all pharmacies in Sweden since 2005. The HbA1c register covers the population in the city of Malmö. Many cases were detected during repeated surveys of the MDC cohort. However, diabetes can often go undetected for a long time and cases that did not seek medical care might have been missed.

Changes in exposure are an inherent problem in long-term cohort studies. It is possible that cadmium levels changed during the 14 years follow-up. However, the body burden of cadmium has very long biological half-life (e.g. 10–30 years) [1], [3], [6], [31]. If anything, changes over time should bias the estimates towards the null.

## Conclusions

Elevated blood cadmium levels are not associated with increased incidence of diabetes. The positive association between HbA1c and blood cadmium levels has a likely explanation in mechanisms related to erythrocyte turnover and smoking.
